# Benefits of Wasabi Supplements with 6-MSITC (6-Methylsulfinyl Hexyl Isothiocyanate) on Memory Functioning in Healthy Adults Aged 60 Years and Older: Evidence from a Double-Blinded Randomized Controlled Trial

**DOI:** 10.3390/nu15214608

**Published:** 2023-10-30

**Authors:** Rui Nouchi, Natasha Y. S. Kawata, Toshiki Saito, Haruka Nouchi, Ryuta Kawashima

**Affiliations:** 1 Department of Cognitive Health Science, Institute of Development, Aging and Cancer (IDAC), Tohoku University, Seiryo-machi 4-1, Sendai 980-8575, Japan; haruka.nouchi.e8@tohoku.ac.jp (H.N.); ryuta@tohoku.ac.jp (R.K.); 2 Smart Aging Research Center (S.A.R.C.), Tohoku University, Seiryo-machi 4-1, Sendai 980-8575, Japan; 3 School of Psychological Sciences, University of Human Environment, Dodohimata 9-12, Matsuyama 790-0823, Japan; 4 Department of Functional Brain Imaging, Institute of Development, Aging and Cancer (IDAC), Tohoku University, Seiryo-machi 4-1, Sendai 980-8575, Japan; nkawata@u-fukui.ac.jp (N.Y.S.K.); t.saito18@kurenai.waseda.jp (T.S.); 5 Research Center for Child Mental Development, University of Fukui, Fukui 910-1193, Japan; 6 School of Fundamental Science and Engineering, Waseda University, Tokyo 169-8050, Japan; 7 Japan Society for the Promotion of Science, Tokyo 102-0083, Japan

**Keywords:** wasabi, 6-MSITC, Hexaraphane, isothiocyanate, memory function, nutrition intervention

## Abstract

Background: Cognitive functions decline with age. Declined cognitive functions negatively affect daily behaviors. Previous studies showed the positive effect of spices and herbs on cognition. In this study, we investigated the positive impact of wasabi, which is a traditional Japanese spice, on cognitive functions. The main bioactive compound of wasabi is 6-MSITC (6 methylsulfinyl hexyl isothiocyanate), which has anti-oxidant and anti-inflammatory functions. Anti-oxidants and anti-inflammatories have an important role in cognitive health. Therefore, 6-MSITC is expected to have positive effects on cognitive function. Previous studies showed the beneficial effects on cognitive functions in middle-aged adults. However, it is unclear that 6-MSITC has a positive effect on cognitive functions in healthy older adults aged 60 years and over. Here, we investigated whether 12 weeks’ 6-MSITC intervention enhances cognitive performance in older adults using a double-blinded randomized controlled trial (RCT). Methods: Seventy-two older adults were randomly assigned to 6-MSITC or placebo groups. Participants were asked to take a supplement (6-MSITC or a placebo) for 12 weeks. We checked a wide range of cognitive performances (e.g., executive function, episodic memory, processing speed, working memory, and attention) at the pre- and post-intervention periods. Results: The 6-MSITC group showed a significant improvement in working and episodic memory performances compared to the placebo group. However, we did not find any significant improvements in other cognitive domains. Discussion: This study firstly demonstrates scientific evidence that 6-MSITC may enhance working memory and episodic memory in older adults. We discuss the potential mechanism for improving cognitive functions after 6-MSITC intake.

## 1. Introduction

Cognitive performances decline with age [1]. Older adults show lower cognitive performances compared to young adults. Furthermore, cognitive decline affects daily behavior. For example, older adults with lower cognitive performances feel difficulties in daily behaviors such as shopping, banking, and cooking [2]. Therefore, it is important to improve cognitive functions in older adults.

Nutrition is an important factor for cognitive health in older adults [3,4]. For example, specific dietary patterns (e.g., the Mediterranean diet) have positive effects on cognitive functions such as memory functions and global cognitions [4]. Additionally, several studies using systematic reviews have shown that vegetable and fruit intakes lead to improved cognitive functions as well as brain functions in healthy older adults [5].

Recent studies have also demonstrated that spices and herbs have health benefits [6,7,8]. For example, ginger and garlic intake improve cognitive functions in older adults with and without dementia [8,9]. Several spices and herbs can be widely used in daily dishes [10]. For example, we use them in cooking for flavoring, masking, and coloring [11]. Therefore, testing the positive impact of spices and herbs on cognition has attracted great attention.

Wasabi (Eutrema japonicum) is a Japanese traditional spice. In Japan, wasabi is popular. The main bioactive compound of wasabi is Hexaraphane (6 methylsulfinyl hexyl isothiocyanate: 6-MSITC), which is an isothiocyanate family [12], and has anti-oxidant and anti-inflammatory functions [13,14]. Previous studies suggested that anti-oxidants and anti-inflammatories have an important role in cognitive health in older adults [15,16,17,18]. Therefore, 6-MSITC is expected to have a positive effect on cognitive performances in older adults.

Only two studies investigated beneficial effects of 6-MSITC on cognitive functions [19,20]. Previous studies reported beneficial effects of 6-MSITC on cognitive functions in middle-aged adults (average age = 56 years old) with subjective memory complaints [20] and in the middle-aged adults (average age = 37.5 years old) with chronic fatigue syndrome [19]. For example, a previous study, using a randomized control trial (RCT) for middle-aged adults with subjective memory complaints, reported that the 6-MSITC-intake group improved executive functions measured by the Stroop test compared to the placebo groups. In addition, an open-label trial study for myalgic encephalomyelitis/chronic fatigue syndrome showed significant improvements in processing speed performances measured by the Trail making test A [19]. These results indicate that 6-MSITC intake would lead to improved cognitive performances such as executive functions and processing speed in middle-aged adults with subjective memory complaints and in middle-aged patients with chronic fatigue syndrome. However, it is still unclear whether 6-MSITC intake would have beneficial effects on cognitive functions in healthy older adults.

In this study, we tested whether 12 weeks’ 6-MSITC intake would enhance cognitive performances in older adults. In this study, we made three main hypotheses. First, we hypothesized that 6-MSITC would improve episodic memory as well as working memory performances in older adults because a previous study on patients using 6-MSITC reported a significant improvement of the subjective severity of brain fog symptoms [19]. A general memory complaint is a main symptom of brain fog [21]. Therefore, we additionally hypothesized that, as one human study has reported that Sulforaphane (4-methylsulfinyl butyl isothiocyanate (4-MSITC)), which is the same isothiocyanate (ITC) group as 6-MSITC, would enhance working memory in healthy older adults [22]. Second, we assumed that 6-MSITC intake would also improve inhibition performances. A previous human study showed that 6-MSITC improved inhibition performances measured by the Stroop test in the subjective memory complaints of middle-aged adults [20]. Third, we hypothesized that 6-MSITC would improve processing speed performances in healthy older adults. A previous study, using 6-MSITC, showed significant improvements in processing speeds in middle-aged patients with chronic fatigue [19]. In addition, previous studies, using Sulforaphane, showed improvements in processing speeds in healthy older adults [22,23]. We investigated these hypotheses using a double-blinded randomized controlled trial (RCT) in healthy older adults.

## 2. Materials and Methods

### 2.1. Setting of Trial

This RCT was conducted in Sendai from October 2018 to March 2019. The study was approved by the Tohoku University Hospital Ethical Committee. This study was registered at the University Hospital Medical Information Network (UMIN) Clinical Trial Registry (UMIN 000032694).

**Figure 1:**
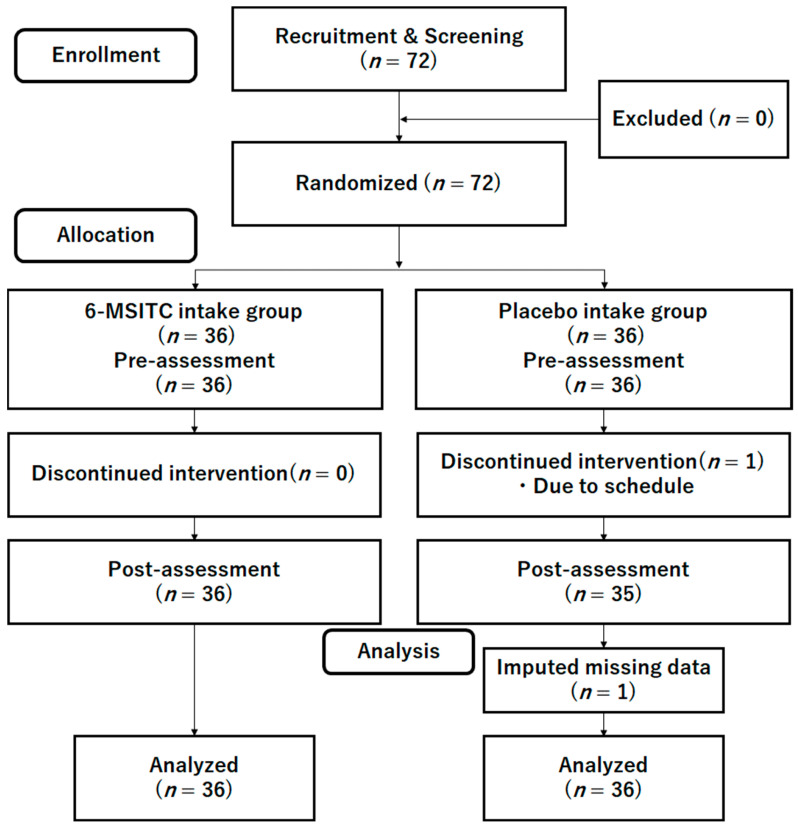
CONSORT diagram.

We investigated the positive effects of 6-MSITC on cognitive performances using a double-blinded RCT (Figure 1). Participants and testers did not know the study hypothesis. Researchers, participants, and testers did not know whether they took the 6-MSITC supplement or the placebo supplement. The primary outcome was cognitive functions. This study was based on the Consolidated Standards of Reporting Trials (CONSORT) statement (see Supplementary Material S1).

### 2.2. Participants

To recruit participants, we posted advertisements in the local town paper in Sendai. The exclusion and inclusion criteria were written in the advertisements. Firstly, researchers checked whether the interested participants met eligibility based on the criteria, such as their basic information, medical history, and food allergies (please see Section 2.3). During the orientation meeting, the researcher (R.N.) explained the study details and received informed consents. Then, all participants took screening assessments (Frontal Assessment Battery at bedside (FAB) [24], the Mini-Mental State Examination (MMSE) [25], and the Geriatric Depression Scale (GDS) [26]). We checked medical histories and physical health by self-reports. No participant was excluded based on the inclusion and exclusion criteria. The participants were randomly assigned to the 6-MSITC or placebo supplement group. During the 12 weeks intervention period, one participant in the placebo group dropped out because of the schedule. Please see Figure 1. The baseline characteristics are shown in Table 1 (average age = 65.43 years (SD = 3.78); 19 males, 53 females).

### 2.3. Inclusion and Exclusion Criteria

Based on previous studies [23], we set the inclusion criteria as follows: (1) right-handed native Japanese speakers without food allergies; (2) 60–80 years of age; (3) not using medications known to interfere with cognitive functions; (4) no history of mental disorders, diabetes, cranial nerve disease, and cardiac disease; and (5) non-heavy alcoholic drinker a day (less than three bottles of beer). Participants with lower cognitive functions (MMSE < 27, FAB < 13) and higher depressive moods (GDS > 4) were excluded. In addition, participants who participated in other intervention studies related to cognitive improvements within 2 months were also excluded.

### 2.4. Sample Size Estimation

The sample size was calculated by G power [27]. The sample size was estimated using the previous results using ITC intervention [20,22]. A previous study using 12 weeks’ SFN 12 improved the working memory capacity of older adults (f = 0.24) [22]. Additionally, an 8 weeks’ 6-MSITC intervention reported a large effect size (f = 0.36) in middle-aged adults with memory complaints [20]. Thus, we expected a medium-to-large effect size (f = 0.34). We used an analysis of covariance (ANCOVA) model. In this model, the covariates were age, sex, and pre-intervention cognitive function score (MMSE). We used the following settings: 0.80 power and α = 0.05. We estimated about a 3% dropout rate in this study based on previous studies [20,22]. The sample size was 72.

### 2.5. Randomization

We used a randomization program using the Graphpad (version 9) tool. We stratified participants based on sex with 1:1 [28]. In the blocked randomization, we set 6 as the block size.

### 2.6. General Procedure of the Intervention

Participants in both groups were asked to take one tablet (6-MSITC or the placebo supplements) before going to bed every day for 12 weeks. Cognitive functions in all participants were assessed at the pre- and post-intervention period. Participants were also required to record their supplement intakes in the diary. After the intervention period, participants returned the remaining supplements and their diaries to the researchers. Then, we confirmed their adherence by checking the dairy and counting the remaining supplements.

Previous studies using the 6-MSITC intervention set the intervention period at 8 weeks [20] or 12 weeks [19]. Recent nutrition intervention studies have reported significant beneficial effects on cognition and brain functions using the 12-week intervention period [22,29,30,31]. Therefore, in this study, we used a 12-week intervention period.

### 2.7. 6-MSITC and Placebo Supplements

The 6-MSITC group took one 6-MSITC capsule that contained 100 mg wasabi extract powder containing 6-MSITC (0.8 mg) absorbed on α cyclodextrin and a 100 mg vehicle (calcium stearate, starch, and silicon dioxide) per day. The placebo group took one placebo capsule containing α 100 mg cyclodextrin and the vehicle per day. The main compositions in each capsule are summarized in Table 2.

We set the dose of 6-MSITC (0.8 mg) based on previous findings using 6-MSITC. There were two reasons. First, the dose of 6-MSITC differed among previous studies (from 0.8 to 9.6 mg). Previous studies with 4.8 mg or 9.6 mg of 6-MSITC were conducted using a pre–post intervention design without control groups [19,32]. However, only one study used the RCT design [20]. This previous RCT study with 0.8 mg of 6-MSITC showed positive effects on cognitive functions in middle-aged adults [20]. Second, a previous safety evaluation study using a five-time overdose revealed that an intake of 0.8 mg of 6-MSITC per day did not have side effects on human health [33]. Based on these findings, we used 0.8 mg of 6-MSITC in this study. Kinjirushi Co., Ltd. (Nagoya, Japan) provided the 6-MSITC and placebo capsules.

### 2.8. Cognitive Functions

We performed the screening tests at the baseline using the Japanese version of MMSE [25] for general cognitive functions, FAB [24] for frontal lobe functions, and JART for general IQs [34].

To measure several cognitive domains (processing speed, attention, short-term memory, working memory, episodic memory, executive functions, and visual–spatial abilities), we used standardized cognitive assessments for healthy older adults. The detail of each test is shown in the Supplemental Information. Processing speed was measured by symbol search (SS) and digit symbol coding (Cd) from the WAIS-III [35]. In SS, participants were asked to judge whether the target symbols were included in a search group. In Cd, participants were asked to draw a specific symbol corresponding to each number (from 0 to 9). Attention performance was evaluated by the digit cancellation task (D-CAT) [36]. In D-CAT, participants were asked to detect the target number in a test sheet. Inhibition was measured by a Stroop task (ST) and a reverse Stroop task (rST) [37]. We used a paper and pencil version of the ST and rST. In the ST, participants were asked to answer the color of the ink of the target (e.g., if “red” was printed in blue ink, then the correct answer was blue color). In the rST, participants were asked to answer the meaning of the target word (e.g., if “red” was printed in blue ink, then the correct answer was red). Colored progressive Matrices (CPMs) tests [38] were used to measure reasoning. In the CPM tests, participants were asked to complete a drawing to select a missing part. Short-term memory was evaluated by digit span forward (DS-F) from the WAIS-III [35]. Working memory was measured by the digit span backward (DS-B) from the WAIS-III [35]. In DS-F and DS-B, participants were asked to memorize a series of digit numbers. Then, participants answered the series of digit numbers in forward (DS-F) or in reverse order (DS-B). For episodic memory, the logical memory (LM) from the WMS-R [39] was used for verbal episodic memory. In LM, participants were required to memorize a story. Then, participants remembered the story immediately (immediate LM) and after 30 min (delayed LM). Additionally, the face and second name test (FSN) was used from the Rivermead Behavioural Memory Test (RBMT) [40]. In FSN, participants were asked to memorize names with faces. The mental rotation test (MR) was used to evaluate visual–spatial performances [41]. In MR, participants were required to select a target figure among rotated figures.

These cognitive assessments are often used in RCTs in healthy older adults [22,23]. Psychological testers with substantial experience of psychological assessments conducted all the cognitive function tests. The psychological testers did not know the current research hypotheses and the group assignments.

### 2.9. Analysis

In this study, we used the intention to treat (ITT) principle. We calculated the change scores in each cognitive function (post–pre difference score). R (ver. 4.10) was used to conduct all analyses. A multiple imputation method with predictive mean matching was used to impute data (m = 20). All variables were included in the data imputation process. The multiple imputations were performed using the function of “mice” of the mice package in R [42]. Then, we performed an analysis of covariance (ANCOVA) with permutation tests, because permutation tests are suitable for non-normal distribution data [43]. In the ANCOVA, the dependent variable was the change scores. The covariates were age, MMSE score, sex, and the pre-scores in the dependent variable. The independent value was the group. All ANCOVAs were performed using the “aovp” function of the lmPerm package in R [44]. Finally, significance was inferred for *p* < 0.05 for multiple comparison methods using the Bonferroni method.

## 3. Results

We did not find any significant difference between the two groups at the baseline (Table 1 and Table 3). The days of supplement intake (max = 84 days) did not differ between the 6-MSITC (average = 83.22, SD = 1.12) and placebo groups (average = 83.31, SD = 1.72).

We performed ANCOVAs for the change scores (Table 4). The 6-MSITC group showed a significant improvement in working memory, measured by DS-B (F (1, 66) = 8.20, η^2^ = 0.11, adjusted *p* = 0.000), and in episodic memory performance, measured by LM immediately (F (1, 66) = 15.80, η^2^ = 0.19, adjusted *p* = 0.00), LM delay (F (1, 66) = 10.39, η^2^ = 0.14, adjusted *p* = 0.03), and FSN (F (1, 66) = 12.65, η^2^ = 0.16, adjusted *p* = 0.00). However, we did not find significant improvements in other cognitive functions, such as processing speed (SS: (F (1, 66) = 0.05, η^2^ = 0.00, non-adjusted *p* = 0.80, adjusted *p* = 1.00), Cd: (F (1, 66) = 0.46, η^2^ = 0.00, non-adjusted *p* = 0.40, adjusted *p* = 1.00)), short-term memory (DS-F: F (1, 66) = 0.03, η^2^ = 0.00, non-adjusted *p* = 0.80, adjusted *p* = 1.00), attention (D-CAT: (F (1, 66) = 0.89, η^2^ = 0.01, non-adjusted *p* = 0.62, adjusted *p* = 1.00), inhibition (ST: F (1, 66) = 0.09, η^2^ = 0.00, non-adjusted *p* = 0.62, adjusted *p* = 1.00), rST: F (1, 66) = 0.75, η^2^ = 0.01, non-adjusted *p* = 0.32, adjusted *p* = 1.00)), reasoning (CPM: F (1, 66) = 0.09, η^2^ = 0.00, non-adjusted *p* = 0.69, adjusted *p* = 1.00), and visuo-spatial performances (MR: F (1, 66) = 0.09, η^2^ = 0.00, non-adjusted *p* = 0.77, adjusted *p* = 1.00).

## 4. Discussion

This study aimed to test whether 12 weeks’ 6-MSITC intervention has positive effects on cognitive performances in healthy older adults. We found two main findings. First, the 6-MSITC intervention improved working memory capacity as measured by DS-B compared to the placebo group. Second, the 6-MSITC intervention improved episodic memory performances as measured by LM and FSM. These findings support our first hypothesis. However, contrary to our second hypothesis, we did not find any significant improvements in inhibition. These findings suggest that the 12 weeks’ 6-MSITC intake selectively enhances working and episodic memory functions in healthy older adults.

In this study, working memory performances, as measured by DS-B, were improved after the 12 weeks’ 6-MSITC intake compared to the placebo intake. This result is consistent with previous studies using Sulforaphane [22]. A recent human study reported that a 12 weeks’ combined intervention using Sulforaphane intake improved working memory performances, as measured DS-B, in older adults [22]. However, this is the first study to demonstrate that 6-MSITC alone enhances working memory capacity in healthy older adults.

The second finding is that the 12 weeks’ 6-MSITC intake improved episodic memory performances, as measured by LM and FSN, in healthy older adults. This finding is consistent with a previous patient study using 6-MSITC in middle-aged patients with chronic fatigue [19]. The previous study reported that 6-MSITC reduced the symptoms of brain fog. The main symptom of brain fog is a subjective memory complaint. However, our finding expands the previous evidence to demonstrate the improvements in different episodic memory test performances using LM and FSN in healthy older adults. LM measured verbal episodic memory performance using a short story. A previous study reported a significant correlation between LM performance and the activities of daily living [45]. On the other hand, FSN is a pair association between faces and names. The difficulty of remembering names is a major memory problem [46]. It is important to improve the memory performance of stories, faces, and names in older adults [47]. Therefore, our finding suggests that 6-MSITC intake would be an effective approach to enhancing daily memory functioning in the aging population.

In contrast to the third and fourth hypotheses, there are no significant changes in the inhibition and processing speed performances after the 6-MSITC intake in older adults. This is inconsistent with a previous study showing a beneficial effect of 6-MSITC on inhibition performance [20]. But there are some differences in methods between this study and previous studies [19,20], such as the intervention period (12 weeks or 8 weeks), subjective cognitive complaints (healthy, subjective memory complaints, patients with chronic fatigue), psychological tests (Trail making test, SS, or Cd), and participants’ age (older adults or middle-aged adults). Therefore, it would not be possible to conclude that there are benefits of 6-MSITC on inhibition and processing speed performances. To discuss the benefits of 6-MSITC on inhibition and processing performances, further studies are needed.

It is important to consider a potential mechanism of the improvements in memory functions after 6-MSITC intake. Previous studies have demonstrated that 6-MSITC has anti-oxidant and anti-inflammatory functions [13,14]. These functions are important for increasing cognitive functions in older adults [15,16]. The hippocampus has a critical role in working and episodic memory performances [48]. Taken together, we hypothesized the following mechanism. First, 6-MSITC would reduce oxidant and inflammatory levels in the hippocampus. The decrease of oxidants and inflammation may protect brain damage and enhance neural functions such as brain activities and neural plasticity in the hippocampus. The hippocampus is important for memory functioning. Therefore, 12 weeks’ 6-MSITC intake would enhance memory functioning in older adults.

This study has some limitations. First, we did not measure any biomarkers of anti-oxidants or anti-inflammatories. To understand the biological mechanism of the benefit of 6-MSITC, it is important to investigate a change in the biomarkers before and after intervention periods. Second, we recruited only healthy older adults. To generalize the current findings, further investigation is needed on whether 6-MSITC would improve cognitive functions in young adults. Third, the number of female participants was larger than that of the male participants in this study, even though we recruited participants from the public using advertisements. We used sex as the covariate in all analyses. Therefore, the effect of sex would be reduced in this study. However, it is important to perform RCTs with a 1:1 sex ratio in future studies.

## 5. Conclusions

6-MSITC is the main bioactive compound of wasabi. We investigated the benefits of 6-MSITC intake on cognitive health in older adults. The current RCT revealed that an intake of 0.8 mg of 6-MSITC for 12 weeks significantly improved memory functioning, including episodic and working memory, compared to the placebo group, but we did not find any significant improvements in other cognitive functions. This study is the first to demonstrate that 6-MSITC has a benefit on memory functioning in healthy older adults.

## Figures and Tables

**Table 1:** Baseline data in both groups.

	6-MSITC (Female: N = 9, Male: N = 27)		Placebo (Female: N = 10, Male: N = 26)			
	Mean	SD	Mean	SD	*p* Value	d
Age	65.92	3.94	64.94	3.61	0.28	0.26
Education (years)	12.5	0.8	12.9	1.1	0.87	0.04
MMSE	28.5	1.48	28.75	1.57	0.49	0.16
FAB	15.39	1.86	15.37	1.93	0.79	0.06
JART	20.33	3.85	20.78	3.62	0.62	0.12
GDS	2.44	1.36	2.07	1.07	0.18	0.31

Note: 6-MSITC, 6 mthylsulfinyl hexyl isothiocyanate; MMSE, Mini-Mental State examination; FAB, Frontal Assessment Battery at bedside; JART, Japanese Reading Ability Test; GDS, Geriatric Depression Scale; d, Cohen’s d (effect size).

**Table 2:** Compositions in each capsule.

Main Ingredients	6-MSITC Capsule	Placebo Capsule
wasabi extract powder (contains 0.8 mg of 6-MSITC)	22.40%	0%
α cyclodextrin	77.60%	100%

Note: 6-MSITC, 6 mthylsulfinyl hexyl isothiocyanate.

**Table 3:** Cognitive functions of both groups at the baseline.

	6-MSITC		Placebo			
	Mean	SD	Mean	SD	*p* Value	d
Processing speed						
Cd	75.89	10.16	76.42	9.19	0.82	0.05
SS	38.58	5.96	37.17	4.99	0.28	0.26
Attention						
D-CAT	48.69	7.55	51.19	8.08	0.18	0.32
Executive functions						
ST	34.75	6.33	36.17	5.82	0.33	0.23
rST	48.97	6.06	48.69	6.71	0.85	0.04
Reasoning						
CPM	34.5	1.65	33.86	3.55	0.33	0.23
Short-term memory						
DS-F	8.36	1.84	8.97	2.34	0.22	0.29
Working memory						
DS-B	6.42	1.96	7.06	2.76	0.26	0.27
Episodic memory						
Immediate LM	12.03	3.85	12.47	3.18	0.6	0.13
Delayed LM	10.89	4.16	11.61	3.87		
FSN	4.39	2.31	4.67	1.67	0.56	0.14
Visual–spatial performance						
MR	19.56	5.46	18.78	4.61	0.52	0.15

Note: 6-MSITC, 6 mthylsulfinyl hexyl isothiocyanate; SD, standard deviation; Cd, digit symbol coding; SS, symbol search; D-CAT, digit cancellation task; ST, Stroop task; rST, reverse Stroop task; CPM, Raven’s colored progressive matrices; DS-F, digit span forward; DS-B, digit span backward; LM, logical memory; FSN, face and second name test; MR, mental rotation test; d, Cohen’s d (effect size).

**Table 4:** Change scores of cognitive functions of both groups.

	6-MSITC		Placebo			Adjusted *p* Value ^1^	
	Mean	SD	Mean	SD	*p* Value		Eta^2^
Processing speed							
Cd	1.72	5.19	2.37	7.15	0.40	1.00	0.01
SS	0.86	3.71	0.86	3.6	0.80	1.00	0.00
Attention							
D-CAT	1.22	6.83	1.11	7.44	0.65	1.00	0.00
Executive functions							
T	1.28	3.27	0.69	4.93	0.62	1.00	0.00
rST	0.61	4.15	0.23	3.99	0.32	1.00	0.01
Reasoning							
CPM	0.31	1.43	0.31	1.71	0.69	1.00	0.00
Short-term memory							
DS-F	0.14	1.46	0.29	1.49	0.80	1.00	0.00
Working memory							
DS-B	1.14	1.84	−0.03	1.98	0.00	0.00	0.11
Episodic memory							
Immediate LM	1.53	2.66	−0.11	2.68	0.00	0.00	0.19
Delayed LM	1.97	3.28	0.34	3.03	0.00	0.03	0.14
FSN	1.78	2.03	0.69	1.95	0.00	0.00	0.16
Visual–spatial performance							
MR	−0.28	6.97	0.37	4.38	0.76	1.00	0.00

Note: 6-MSITC, 6 mthylsulfinyl hexyl isothiocyanate; SD, standard deviation; Cd, digit symbol coding; SS, symbol search; D-CAT, digit cancellation task; ST, Stroop task; rST, reverse Stroop task; CPM, Raven’s colored progressive matrices; DS-F, digit span forward; DS-B, digit span backward; LM, logical memory; FSN, face and second name test; MR, mental rotation test. ^1^ *p* values were adjusted by the Bonferroni method.

## Data Availability

The datasets used and analyzed in this study are available from the corresponding author on reasonable request.

## Supplementary Materials

The following supporting information can be downloaded at: https://www.mdpi.com/article/10.3390/nu15214608/s1, Files S1: Details of the cognitive function measures; Table S1: CONSORT checklist.

## Trial Registration

This trial was registered at The University Hospital Medical Information Network Clinical Trials Registry (UMIN 000032694). Registered 24 May 2018, https://upload.umin.ac.jp/cgi-open-bin/ctr/ctr_view.cgi?recptno=R000037285.
