# Supplementary material for: Benefits of Wasabi Supplements with 6-MSITC (6-Methylsulfinyl Hexyl Isothiocyanate) on Memory Functioning in Healthy Adults Aged 60 Years and Older: Evidence from a Double-Blinded Randomized Controlled Trial
Source: Nutrients. 2023 Oct 30;15(21):4608. doi: 10.3390/nu15214608 (PMC10648564; doi:10.3390/nu15214608)
Supplement: Supplementary file 1 [file nutrients-15-04608-s001.zip › Supplementary File S1.pdf]

Details of cognitive functional measures

To assess processing speed, we used digit symbol coding (Cd) and symbol search (SS) from the WAIS-III (Wechsler, 1997). The following descriptions of Cd and SS were reproduced from our earlier report (Nouchi et al., 2012a). "For Cd, the participants were shown a series of symbols that were paired with numbers. Using a key within a 120 s time limit, participants drew each symbol under its corresponding number. The primary measure of this test was the number of correct answers. In SS, participants visually scanned two groups of symbols (a target group and a search group) and indicated whether either of the target symbols matched any symbol in the search group. Participants responded to as many items as possible within a 120 s time limit. The primary measure of this test was the number of correct answers."

To measure attention performance, we conducted the digit cancellation task (D-CAT). The following descriptions of the D-CAT are reproduced from our earlier report (Nouchi et al., 2013). "The test sheet consists of 12 rows of 50 digits. Each row contains five sets of numbers 0–9 arranged in random order. Thus, any one digit would appear five times in each row with randomly determined neighbors. The D-CAT consists of three such sheets. Participants were instructed to search for the target number(s) that had been specified to them and to delete each one with a slash mark as quickly and as accurately as possible until the experimenter sent a stop signal. Three trials were conducted, first with a single target number (6), second with two target numbers (9 and 4), and third with three (8, 3, and 7). Each trial was given 1 min. Consequently, the total time required for D-CAT was 3 min. For the second and third trials, it was emphasized that all instructed target numbers should be canceled without omission. The primary measure of this test was the number of hits (correct answers). We used only the number of hits in the first trial."

To measure inhibition ability of executive functions, we used a Stroop task (ST) and a reverse Stroop task (rST) (Hakoda & Watanabe, 2004) ("In the ST, in the leftmost of six columns, a word naming a color was printed in another color (e.g., "red" was printed in blue letters); the other five columns contain words naming colors. Participants were required to check the column containing the word naming the color of the word in the leftmost column. In the reverse ST, in the leftmost of six columns, a word naming a color was printed in another color (e.g., "red" was printed in blue letters); the other five columns were filled respectively, with five different colors, from which participants were required to check the column with the color matching the written word in the leftmost column. In each task, participants were instructed to complete as many of these exercises

as possible in 1 min. The primary measure for this task was the number of correct items" (Nouchi et al., 2012a).

To measure short-term memory and working memory performance, we used the digit span forward (DS-F) and the digit span backward (DS-B) tasks, which are the subtests of the WAIS-III (Wechsler, 1997). The following descriptions of the DS-F and the DS-B are reproduced from our earlier report (Nouchi et al., 2012a). "For the DS-F, participants repeated numbers in the same order as they were read aloud by the examiner. For the DS-B, participants repeated numbers in the reverse order of that presented aloud by the examiner. In both tasks, the examiner read a series of number sequences which the participant was required to repeat in either forward or reverse order." The primary measures of this test were the digit number length. The maximum digit number length in the DS-F was 8, and that of the DS-B was 7.

To measure episodic memory, we used the logical memory (LM) subtest of the WMS-R (Wechsler, 1987) and FSN is a subset of Rivermead Behavioural Memory Test (RBMT)(Wilson et al., 1985): "LM consists of two short-paragraph-length stories (Story A and Story B). For the LM activity, participants were required memorize one of the two stories. The stories were scored in terms of the number of story units recalled, as specified in the WMS-R scoring protocol. We used either Story A or Story B. The primary measure for this task was the number of correct story units recalled." (Nouchi et al., 2012a). We checked the LM performances of immediate recall and delayed recall memory. "In FSN, participants must memorize first and second names with faces (photographs). Subsequently, they must recall the first and the second names when the face is shown again later. We use four faces (four first names and four second names). The primary measure of this test is the total number of correct answers in both first and second names. The maximum raw score of FSN is 8." (Nouchi et al., 2012b)

To measure reasoning, we used Raven's Coloured Progressive Matrices (CPM) tests (Raven JC, 1965; Smits et al., 1997). The CPM has 36 items. Each item is printed with a brightly color. The item consists in a drawing with a missing part. Participants were asked to complete the item by choosing one answer among six choices. The maximum score is 36.

To measure visuo-spatial ability, we used mental rotation test (MR) (Peters et al., 1995). The mental rotation test uses three-dimensional cubes figures. The mental rotation test has 24 items. Each item consists of a row of five drawings, a target figure in the left-most position followed by four response-choice figures. The participants were asked to find two choice figures are rotated reproductions of the target figure.

Reference

Hakoda, Y., & Watanabe, M. (2004). *Manual for New Stroop test II*. Toyo Physical.

Nouchi, R., Nouchi, H., & Kawashima, R. (2020). A Single 30 Minutes Bout of Combination Physical Exercises Improved Inhibition and Vigor-Mood in Middle-Aged and Older Females: Evidence From a Randomized Controlled Trial. *Frontiers in Aging Neuroscience*, *12*, 179. https://doi.org/10.3389/fnagi.2020.00179

Nouchi, R., Taki, Y., Takeuchi, H., Hashizume, H., Nozawa, T., Kambara, T., Sekiguchi, A., Miyauchi, C. M., Kotozaki, Y., Nouchi, H., & Kawashima, R. (2013). Brain training game boosts executive functions, working memory and processing speed in the young adults: a randomized controlled trial. *PloS One*, *8*(2), e55518. https://doi.org/10.1371/journal.pone.0055518

Nouchi, R., Taki, Y., Takeuchi, H., Hashizume, H., Nozawa, T., Sekiguchi, A., Nouchi, H., & Kawashima, R. (2012a). Beneficial effects of reading aloud and solving simple arithmetic calculations (learning therapy) on a wide range of cognitive functions in the healthy elderly: study protocol for a randomized controlled trial. *Trials*, *13*(1), 32. https://doi.org/10.1186/1745-6215-13-32

Nouchi, R., Taki, Y., Takeuchi, H., Hashizume, H., Nozawa, T., Sekiguchi, A., Nouchi, H., & Kawashima, R. (2012b). Beneficial effects of short-term combination exercise training on diverse cognitive functions in healthy older people: study protocol for a randomized controlled trial. *Trials*, *13*(1), 200. https://doi.org/10.1186/1745-6215-13-200

Peters, M., Laeng, B., Latham, K., Jackson, M., Zaiyouna, R., & Richardson, C. (1995). A redrawn vandenberg and kuse mental rotations test - different versions and factors that affect performance. *Brain and Cognition*, *28*(1), 39–58. https://doi.org/10.1006/brcg.1995.1032

Raven JC. (1965). *Guide to using the coloured progressive matrice: Sets A. Ab. B.1*. Lewis.

Smits, C. H. M., Smit, J. H., van den Heuvel, N., & Jonker, C. (1997). Norms for an abbreviated Raven's Coloured Progressive Matrices in an older sample. *Journal of Clinical Psychology*, *53*(7), 687–697.

Wechsler, D. A. (1987). *Wechsler Memory Scale Revised*. The Psychological Corporation.

Wechsler, D. A. (1997). *Wechsler Adult Intelligence Scale Third edition*. The Psychological Corporation.

Wilson, B. A., Cockburn, J., & Baddeley, A. D. (1985). *The Rivermead Behavioral Memory Test.* Thamas Valley Test Company.
